# Evaluation of Xpert point-of-care assays for detection of HIV infection in persons using long-acting cabotegravir for pre-exposure prophylaxis

**DOI:** 10.1128/spectrum.00307-24

**Published:** 2024-07-09

**Authors:** Jessica M. Fogel, Estelle Piwowar-Manning, Amber Moser, Tinia Hill, Shahnaz Ahmed, Vanessa Cummings, Heba H. Mostafa, Zhe Wang, Andrea Jennings, Jorge A. Gallardo-Cartagena, María Inés Figueroa, Marty St Clair, Alex R. Rinehart, Adeola Adeyeye, James F. Rooney, Myron S. Cohen, Beatriz Grinsztejn, Raphael J. Landovitz, Susan H. Eshleman, On behalf of the HPTN 083 Study Team

**Affiliations:** 1 Department of Pathology, Johns Hopkins University School of Medicine, Baltimore, Maryland, USA; 2 Fred Hutchinson Cancer Center, Seattle, Washington, USA; 3 FHI 360, Durham, North Carolina, USA; 4 Centro de Investigaciones Tecnológicas, Biomédicas y Medioambientales (CITBM), Universidad Nacional Mayor de San Marcos, Lima, Peru; 5 Clinical Research Department, Fundación Huésped, Buenos Aires, Argentina; 6 ViiV Healthcare, Research Triangle Park, North Carolina, USA; 7 Prevention Science Program, Division of AIDS, National Institute of Allergy and Infectious Diseases, National Institutes of Health, Rockville, Maryland, USA; 8 Gilead Sciences, Foster City, California, USA; 9 Department of Medicine, University of Carolina, Chapel Hill, North Carolina, USA; 10 Instituto de Pesquisa Clinica Evandro Chagas-Fiocruz, Rio de Janeiro, Brazil; 11 Center for Clinical AIDS Research & Education, University of California, Los Angeles, California, USA; University of Miami, Miami, Florida, USA

**Keywords:** HIV prevention, long-acting cabotegravir, HPTN 083, pre-exposure prophylaxis, point-of-care, RNA

## Abstract

Detection of HIV infection may be challenging in persons using long-acting cabotegravir (CAB-LA) pre-exposure prophylaxis (PrEP) due to viral suppression and reduced/delayed antibody production. We evaluated two point-of-care tests for detecting HIV infection in persons who received CAB-LA in the HPTN 083 trial. Samples were obtained from 12 participants who received CAB-LA and had delayed detection of HIV infection using HIV rapid tests and an antigen/antibody test (52 plasma samples; 18 dried blood spot [DBS] samples). Plasma samples were tested with the Xpert HIV-1 Viral Load XC test (Xpert VL-XC); DBS samples were tested with the total nucleic acid Xpert HIV-1 Qual XC test (Xpert Qual-XC). Results from these assays were compared to results from three reference, laboratory-based, plasma RNA assays (Aptima HIV-1 Qualitative assay [Aptima Qual]; Aptima HIV-1 Quant DX Assay [Aptima Quant]; cobas HIV-1/HIV-2 Qualitative Test [cobas]). HIV RNA was detected with all four plasma assays for all samples with viral loads (VLs) ≥ 200 copies/mL; the number of samples with VLs < 200 copies/mL with HIV RNA detected was: Xpert VL-XC: 19/26 (73.1%); Aptima Qual: 17/26 (65.4%); Aptima Quant: 17/26 (65.4%); and cobas: 12/21 (57.1%). The Xpert Qual-XC assay was positive for all DBS samples with VLs ≥ 200 copies/mL and 1/10 DBS with VLs < 200 copies/mL. The performance of the Xpert VL-XC assay was comparable to the reference assays for detecting HIV infection in these cases. The Xpert Qual-XC assay was less sensitive than plasma-based HIV RNA assays for detecting HIV in the setting of CAB-LA PrEP.

**IMPORTANCE:**

HIV RNA assays can detect HIV infections earlier than HIV rapid tests or Ag/Ab tests in persons using CAB-LA PrEP. Earlier HIV diagnosis could allow for earlier treatment initiation and reduced risk of INSTI resistance. POC tests may help detect HIV infection before CAB-LA administration and may be more accessible than laboratory-based assays in some settings. In this study, the POC Xpert VL-XC assay detected HIV RNA in most samples from individuals who received CAB-LA PrEP and had delayed detection of HIV infection with HIV rapid tests and an Ag/Ab test. The performance of this assay was similar to laboratory-based HIV RNA assays in this cohort. The POC Xpert Qual-XC assay detects both HIV RNA and DNA, with a higher viral load cutoff for RNA detection. This assay was negative for most lower viral load samples and did not offer an advantage for HIV screening in persons using CAB-LA PrEP.

## INTRODUCTION

Long-acting cabotegravir (CAB-LA) is highly effective for HIV prevention (1–3). The United States (US) Food and Drug Administration (FDA) approved CAB-LA for the prevention of sexual acquisition of HIV in 2021 (4); other countries have also received regulatory approvals for the use of CAB-LA for HIV pre-exposure prophylaxis (PrEP) (5).

HIV diagnosis can be challenging in persons using CAB-LA PrEP due to suppression of viral replication and diminished/delayed antibody production (6). HIV RNA levels are often low or undetectable in early infection and may remain low or undetectable for months, even after injections stop and drug levels decline (6). In the HIV Prevention Trials Network (HPTN) 083 study, detection of HIV infections in persons randomized to receive CAB-LA was often delayed when HIV rapid tests and an antigen/antibody (Ag/Ab) test were used for HIV screening (7, 8).

Delayed HIV diagnosis in persons using CAB-LA can lead to the emergence of integrase strand transfer inhibitor (INSTI) resistance; delayed initiation of antiretroviral treatment, and continued administration of CAB-LA after infection (7–10). Prior studies show that HIV RNA screening often detects HIV infections earlier than HIV rapid tests or Ag/Ab tests in persons using CAB-LA (7–10). The use of a point-of-care (POC) HIV nucleic acid test for HIV screening may be especially useful in this setting since it might allow for detection of infection prior to CAB-LA injection.

We evaluated the performance of two POC assays for detection of HIV infection in samples from participants who received CAB-LA in HPTN 083: the Xpert HIV-1 Viral Load XC test (Xpert VL-XC) and the Xpert HIV-1 Qual XC test (Xpert Qual-XC). Results from these assays were compared with those obtained using FDA-cleared, laboratory-based HIV RNA assays.

## MATERIALS AND METHODS

### HPTN 083 study procedures

**Fig 1:**
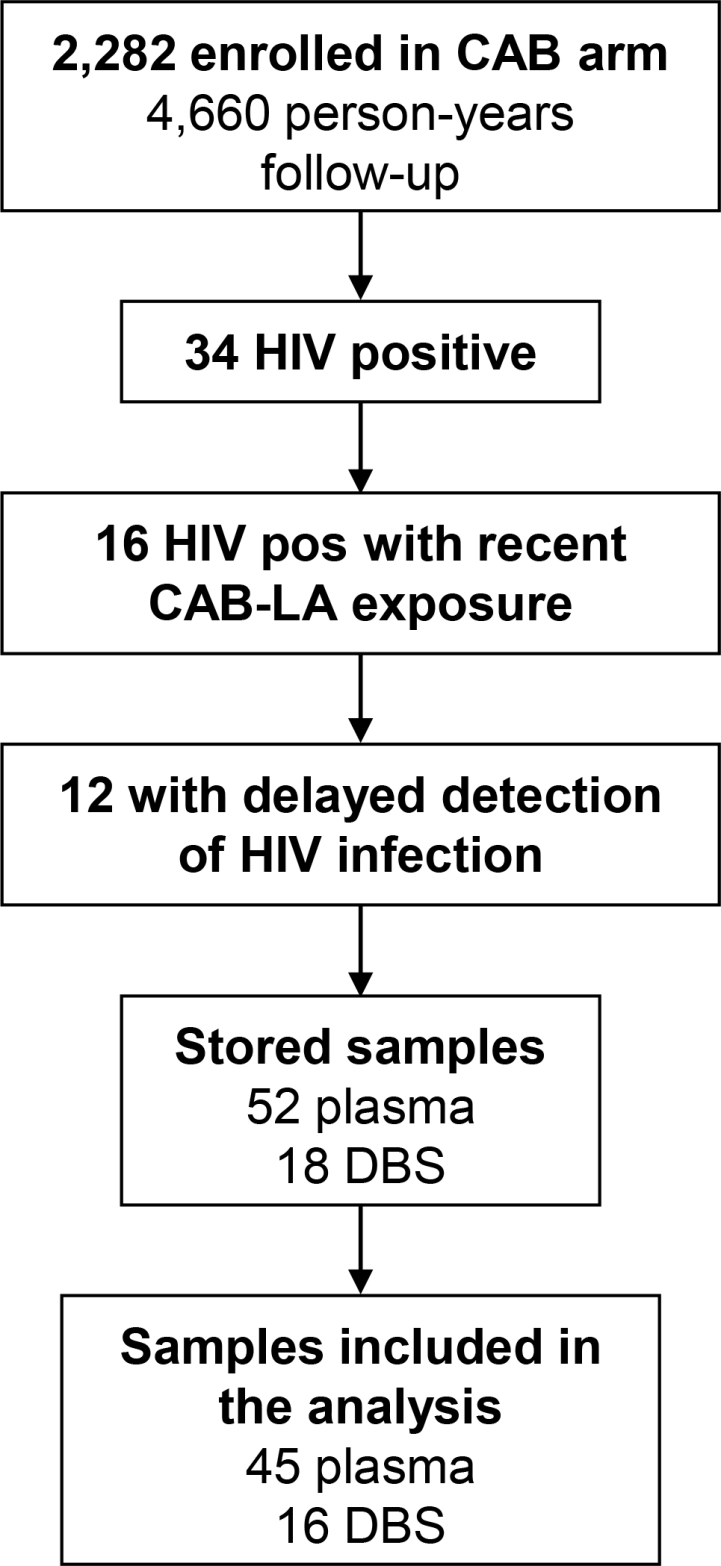
The figure shows the approach used to select samples for testing. In HPTN 083, 2,282 participants were randomized to the cabotegravir (CAB) arm. These participants were followed for 3,204 person-years during the blinded phase of the trial and 1,456 person-years during the first year after study unblinding (total: 4,660 person-years). Thirty-four HIV infections were identified between the start of the trial and the end of the first unblinded year. Sixteen of these participants acquired HIV within 6 months of a long-acting CAB (CAB-LA) injection. In 12 of those cases, detection of HIV infection was delayed at the study site using HIV rapid tests and an antigen/antibody test. Samples from those 12 cases were used for analysis in this study. The samples from these cases were collected in the HPTN 083 trial for Virology and Pharmacology assessments related to the study objectives. This study used plasma samples remaining after those assessments were completed. Some samples did not have sufficient plasma for testing with all of the assays evaluated in this report. Fifty-two plasma samples and 18 dried blood spot (DBS) samples were stored for these 12 participants on and after the first HIV-positive visit. Cases were excluded from the analysis if results were obtained for only one or two of the four plasma assays. The final sample set included 45 plasma and 16 DBS (see File S2). Abbreviations: CAB: cabotegravir; CAB-LA: long-acting injectable CAB; DBS: dried blood spots; pos: positive.

HPTN 083 enrolled cisgender men and transgender women who have sex with men (https://www.clinicaltrials.gov/study/NCT02720094, Fig. 1) (1). Plasma samples were stored at every visit; dried blood spot (DBS) samples were stored at selected visits (1). Methods used to identify and characterize HIV infections in the trial are reported previously (7, 8). US FDA-cleared HIV rapid tests and a laboratory-based Ag/Ab test were used for screening at study sites (1). Cases with reactive test results were analyzed retrospectively at the HPTN Laboratory Center using assays that included the Architect HIV Ag/Ab Combo assay (Ag/Ab test) and the APTIMA HIV-1 RNA Qualitative assay (Aptima Qual; File S1) (7). An independent committee reviewed all HIV test results, determined HIV status, and identified the first HIV-positive visit. The first site-positive visit was the first visit near the time of infection where the study site obtained a reactive/positive HIV test result. The participants selected for inclusion in this study acquired HIV infection within 6 months of CAB-LA injection and had delayed detection of HIV infection at the study site (Fig. 1).

### Additional testing

Additional testing was performed using two POC tests, the Xpert VL-XC (RNA assay) and the Xpert Qual-XC (total nucleic acid assay), and two laboratory-based tests, the Aptima HIV-1 Quant Dx Assay (Aptima Quant) and the cobas HIV-1/HIV-2 Qualitative Test (cobas Qual) (File S1).

## RESULTS

We previously identified 34 participants who received CAB-LA in HPTN 083 (7, 8). This study included 12 of those participants who received CAB-LA within six months of HIV infection and had delayed detection of HIV infection at study sites. Fifty-two plasma samples and 18 DBS samples were available from these 12 participants (median five plasma samples/participant [range: 2–7], median 2 DBS samples/participant [range: 0–3]; Fig. 1). These samples were collected 0–117 days after the first HIV-positive visit; 40 (76.9%) of the 52 plasma samples were collected before the site detected the possibility of infection (File S2). The analysis below includes results from 45 of the 52 plasma samples (five samples had insufficient volume for Xpert VL-XC testing and two samples had Xpert VL-XC result errors). Nineteen of the 45 samples had viral loads (VLs) ≥ 200 copies/mL (higher VL samples; median 3,278 copies/mL, range: 377–222,243, Aptima Quant assay); the remaining 26 had VLs < 200 copies/mL (lower VL samples; median <30 copies/mL [range: not detected-192]; Table 1; File S2). HIV RNA was detected with all four plasma-based assays for all higher VL samples with test results. The number of lower VL samples that had HIV RNA detected for each assay was Xpert VL-XC: 19/26 (73.1%), Aptima Qual: 17/26 (65.4%), Aptima Quant: 17/26 (65.4%), and cobas Qual: 12/21 (57.1%) (Table 1); five of the lower VL samples tested negative with all RNA assays (File S2).

**TABLE 1:** Summary of test results^*a*^

	Aptima Qual^*b*^^,*c*^	Aptima Quant^*b*^	Cobas Qual^*b*^	Xpert VL-XC	Xpert Qual-XC
Assay type	Laboratory-based RNA assay	Laboratory-based RNA assay	Laboratory-based RNA assay	Point-of-care RNA assay	Point-of-care RNA/DNA assay
Sample type	Plasma	Plasma	Plasma	Plasma	DBS
Viral load ≥ 200 c/mL	19/19 (100%)	19/19 (100%)	17/17 (100%)	19/19 (100%)	6/6 (100%)
Viral load < 200 c/mL	17/26 (65.4%)	17/26 (65.4%)	12/21 (57.1%)	19/26 (73.1%)	1/10 (10.0%)

^*a*^ Samples from HPTN 083 were tested with five nucleic acid tests: the APTIMA HIV-1 RNA Qualitative Assay (Aptima Qual), the Aptima HIV-1 Quant Dx Assay (Aptima Quant), the cobas HIV-1/HIV-2 Qualitative Test (cobas Qual), the point-of-care Xpert HIV-1 Viral Load XC test (Xpert VL-XC), and the POC Xpert HIV-1 Qual XC (Xpert Qual-XC). The Xpert Qual-XC assay was performed using dried blood spots (DBS); the other four assays were performed using plasma samples. The table shows the number and percentage of samples with a detected/positive result over the number of samples tested for the five assays evaluated in this report. The number of samples tested with each plasma-based assay varies since some samples did not have sufficient plasma available for testing with all four of these assays.

^*b*^ Approved by the US Food and Drug Administration to aid in the diagnosis of HIV infection.

^*c*^ This assay is no longer available.

Results were obtained with the Xpert Qual-XC assay for 16/18 DBS samples (six with VLs ≥200 copies/mL, 10 with VLs < 200 copies/mL); results for two cases were not included due to missing VL results. HIV was detected in all six DBS samples from participants who had a VL ≥200 copies/mL at that visit, and in 1/10 (10%) of the DBS samples from participants who had a VL <200 copies/mL at that visit (Table 1).

## DISCUSSION

In HPTN 083, only 34 participants in the CAB arm acquired HIV infection over 4,660 person-years of study follow-up (8, 11). Despite the low frequency of infections in this setting, delays in HIV diagnosis due to viral suppression and delayed/diminished antibody production can complicate clinical management and increase the risk of the emergence of INSTI resistance. Sensitive RNA assays can detect infections earlier than HIV rapid tests or Ag/Ab tests in this setting (7, 8, 10, 12). POC nucleic acid tests have the potential to detect infection prior to CAB-LA injection. In low and middle-income settings, POC tests may also facilitate the implementation of RNA screening in persons using CAB-LA PrEP in settings where laboratory-based VL assays are not readily available.

The RNA assays that we evaluated were positive for all plasma samples with higher VLs (≥200 copies/mL) and 57-73% of samples with lower VLs (<200 copies/mL). The POC Xpert VL-XC assay was positive for the greatest number of lower VL samples tested (19/26; 73%); meaningful statistical analysis of these data is not possible due to the small number of cases that met the criteria for inclusion in the study. In contrast, the Xpert Qual-XC assay was negative for most of the samples tested. This likely reflects the higher cutoff for RNA detection with this assay (900 copies/mL) and the low level of HIV DNA often seen in infections that occur in the setting of CAB-LA PrEP (6–8).

While HIV RNA screening can detect some infections earlier than antigen- or antibody-based assays in persons using CAB-LA PrEP, prospective HIV RNA screening may not be feasible or affordable in some settings. False-positive RNA results may also complicate clinical management (13). HIV RNA screening for persons using CAB-LA PrEP is required in the US FDA package insert (14) and guidelines from the US Centers for Disease Control (15) but is not included in testing guidelines from the World Health Organization (16).

The HPTN 083 trial is now in an open-label extension (OLE) phase where participants can choose CAB-LA or daily oral PrEP. In the OLE study, HIV RNA screening is performed prospectively at every injection visit in addition to HIV rapid and Ag/Ab testing. Data from the OLE study will provide important information on the potential advantages and disadvantages of HIV RNA screening in persons using CAB-LA PrEP.

## SUPPLEMENTAL MATERIAL

Supplemental file 1 spectrum.00307-24-s0001.pdf Assays used for testing at the HPTN Laboratory Center.

Supplemental file 2 spectrum.00307-24-s0002.pdf Evaluation of the Xpert VL-XC and Xpert Qual-XC assays.

ASM does not own the copyrights to Supplemental Material that may be linked to, or accessed through, an article. The authors have granted ASM a non-exclusive, world-wide license to publish the Supplemental Material files. Please contact the corresponding author directly for reuse.
