# Supplementary material for: Evaluation of Xpert point-of-care assays for detection of HIV infection in persons using long-acting cabotegravir for pre-exposure prophylaxis
Source: Microbiol Spectr. 2024 Jul 9;12(8):e00307-24. doi: 10.1128/spectrum.00307-24 (PMC11302132; doi:10.1128/spectrum.00307-24)
Supplement: Supplemental file 1 — Assays used for testing at the HPTN Laboratory Center. [file spectrum.00307-24-s0001.pdf]

Supplementary File 1. Assays used for testing at the HPTN Laboratory Center.

The assays described in this report are listed in the table below.

Table 1

**Assay name** (abbreviation)	**Manufacturer**	**Type of assay**	**Sample type**	**LOD or LLOQ**	**Purpose of testing**
Architect HIV Ag/Ab Combo assay (Ag/Ab test)	Abbott Diagnostics, Wiesbaden, Germany	Laboratory-based, Ag/Ab	Plasma	-	Retrospective test performed in the primary study
APTIMA HIV-1 RNA Qualitative Assay[a,b] (Aptima Qual)	Hologic, Marlborough, MA	Laboratory-based, RNA	Plasma	30 c/mL	Retrospective test performed in the primary study and reference test for this study
Aptima™ HIV-1 Quant Dx Assay (Panther platform)[a] (Aptima Quant)	Hologic, Marlborough, MA	Laboratory-based, RNA	Plasma	LLOQ: 30 c/mL<br>LOD: 12 c/mL	Reference test for this study
cobas® HIV-1/HIV-2 Qualitative Test[a,c] (cobas Qual)	Roche Diagnostics Indianapolis, IN	Laboratory-based, RNA	Plasma	12.8-15.4 c/mL (HIV-1)	Reference test for this study
Xpert HIV-1 Viral Load XC (Xpert VL-XC)	Cepheid, Sunnyvale, CA	POC, RNA	Plasma	40 c/mL	Assay under evaluation
Xpert HIV-1 Qual XC (Xpert Qual-XC)	Cepheid, Sunnyvale, CA	POC, RNA/DNA	DBS	900 c/mL[d]	Assay under evaluation

Footnotes for Table 1.
[a] These tests are approved by the United States (US) Food and Drug Administration (FDA) to aid in diagnosis of HIV infection [1].
[b] This assay has been discontinued.
[c] The cobas® 6800 system was used for testing.
[d] The LOD that is shown for the Xpert Qual-XC assay is for HIV RNA detection.

Abbreviations: Ag/Ab: antigen/antibody; c/mL: copies/milliliter; DBS: dried blood spots; LOD: limit of detection; LLOQ: lower limit of quantitation; POC: point-of-care.

Reference

1. Centers for Disease Control and Prevention. FDA approved HIV Tests. Available at: https://www.cdc.gov/hiv/testing/laboratorytests.html.

Sample processing and testing

All of the assays were performed according to the manufacturer's instructions. Additional details are provided below.

Plasma preparation and testing

Plasma was processed at study sites as follows. Briefly, 20 mL tubes of EDTA-anticoagulated whole blood were spun at 800 - 1000 x g for 10 minutes to separate cells and plasma. The plasma was removed and transferred to a sterile centrifuge tube. The plasma was spun again at 800 x g to 1000 x g for 10 minutes to remove any contaminating debris, cells or platelets. Plasma aliquots were stored between -70°C and -90°C. Blood processing and plasma storage were completed within 6 hours of sample collection. Plasma was maintained at -70°C to -90°C prior to shipment to the HPTN Laboratory Center. Specimens were shipped on dry ice and were then stored at -70°C to -90°C prior to testing. This aligns with the kit insert for Aptima Qual and cobas Qual assay which allow extended storage at less than or equal to -60°C for long-term storage. This differs from the kit insert for the Aptima Quant (90 days) and the Xpert VL-XC assay which allows frozen samples to be stored for 6 weeks. Plasma was thawed at 15ºC to 30ºC immediately prior to testing. Most of the aliquots used for testing had no prior freeze thaws. The maximum number of prior freeze thaws was three which was consistent with the package insert for all four plasma assays (see table). The volumes of plasma used for testing are shown in the table. Assays were performed according to the manufacturer's instructions.

Table 2

Assay	Maximum # freeze thaws	Plasma volume used for testing
Aptima Qual	3	500 uL
Aptima Quant	3	700 uL
cobas Qual	3	500 uL
Xpert VL-XC	5	1 mL

DBS processing and testing

DBS were prepared from EDTA-anticoagulated whole blood that was stored at room temperature (approximately 15°C to 25°C) prior to DBS preparation. Sites were instructed to create the DBS within 6 hours of sample collection. Whole blood tubes were inverted 8 times prior to spotting. Twenty-five uL of whole blood was then pipetted directly onto the center of each spot on Whatman Protein Saver Cards (Whatman INC. Schleicher & Schuell) so that the sample was contained within the circle; 5 spots were created for each whole blood sample. The volume used for spotting differed from the kit insert which suggests using a single spot of approximately 60-70 uL. Sites were instructed not to touch, press, or smear the spots. Cards were air dried in a card holder or drying rack for 2-16 hours at room temperature (between 15ºC and 40ºC), avoiding exposure to a fan or heat source. After drying, DBS cards were placed in low gas-permeability plastic bags with a humidity indicator and desiccant pack to reduce humidity. Indicator cards and desiccant packs were stored in the manufacturer's stock airtight containers until cards were dry and ready for freezer storage. Bags containing DBS were stored at -70 to -90°C. Dessicant packs and indicator cards were replaced if a color change was noted that indicated exposure to excessive humidity (40% to 50% or higher). DBS were kept at -70ºC to -90ºC prior to shipping to the HPTN Laboratory Center. Specimens were shipped on dry ice and were then stored at -70°C to -90°C prior to testing. This differs from the kit insert which notes that frozen samples may be stored for up to 16 weeks. DBS were tested with the Xpert Qual-XC assay according to the manufacturer's instructions using one 12-millimeter punch per test. If a sample had a result of "Not detected", testing was repeated using two spots; in all cases, the repeat testing yielded the same result.
