# Supplementary material for: Evaluation of Xpert point-of-care assays for detection of HIV infection in persons using long-acting cabotegravir for pre-exposure prophylaxis
Source: Microbiol Spectr. 2024 Jul 9;12(8):e00307-24. doi: 10.1128/spectrum.00307-24 (PMC11302132; doi:10.1128/spectrum.00307-24)
Supplement: Supplemental file 2 — Evaluation of the Xpert VL-XC and Xpert Qual-XC assays. [file spectrum.00307-24-s0002.pdf]

Supplementary File 2. Evaluation of the Xpert VL-XC and Xpert Qual-XC assays.

This report includes analysis of plasma samples and dried-blood spot (DBS) samples that were obtained from 12 participants in HPTN 083 who had delayed detection of HIV infection at the study site using HIV rapid tests and an antigen/antibody test for HIV screening. Detailed descriptions of these cases are provided in prior publications [1,2]. These cases were classified based on the timing of HIV infection (Table 1).

Table 1

Case identifier	Case type
A2, A3, A4	These participants had HIV infection at the time of study enrollment that was not detected by site testing
C1, C3	These participants acquired HIV infection while they were receiving oral CAB prior to CAB-LA injections
D1-D6	These participants acquired HIV infection despite on-time CAB-LA injections
BR1	The first HIV-positive visit for this participant was at the time of CAB re-initiation, more than 6 months after the last CAB-LA injection

Abbreviations: CAB: cabotegravir; CAB-LA: long-acting injectable cabotegravir.

Table 2 shows the results of real-time testing performed at study sites and retrospective testing performed at the HPTN Laboratory Center. Table 2A shows results from participants who acquired HIV prior to their first CAB-LA injection (A and C cases). Table 2B shows results from participants who acquired HIV infection after their first CAB-LA injection (D and BR cases).

Legend for Tables 2A and 2B.

The tables show the number of days between the first HIV-positive visit (Days since 1st HIV pos) and the sample used for testing. Asterisks indicate visits where the participant received a CAB-LA injection. A description of each of the assays shown in Table 2 is provided in Supplementary File 1. Results from real-time site testing are shown on the left (HIV rapid testing [Rapid Ab], antigen/antibody testing [Ag/Ab test]). Results from retrospective testing performed at the HPTN Laboratory Center are shown on the right. The following assays were performed previously as part of the algorithm for identification and characterization of HIV infections [1]: Ag/Ab test, Aptima Qual assay. The following additional assays were performed for this study: Aptima Qual assay, cobas Qual assay, Xpert VL-XC assay, Xpert Qual-XC assay (see Supplementary File 1). Reactive/positive test results for all assays are shown in bold font. Viral load values are shown as copies/mL. Some samples could not be tested with all four plasma-based assays since they did not have sufficient plasma available for testing (quantity not sufficient, QNS). "Error" indicates that test results were not available due to a technical problem or assay failure. Grey shading indicates that results were obtained for only one or two of the four plasma HIV assays evaluated in this study. Results from those samples are shown in the table but were not used in the analysis of assay performance. Results from samples with viral loads >200 copies/mL are highlighted in green.

Abbreviations: 1st HIV POS: first HIV-positive visit; Ab: antibody; Ag: antigen; ND: not detected; NR: non-reactive; POC: point-of-care; Pos: positive; QNS: quantity not sufficient; R: reactive.

Table 2A

Case	Days since 1st HIV pos	Site test results (real-time)		HPTN Laboratory Center test results (retrospective)					
		Rapid Ab	Ag/Ab test	Ag/Ab test	Aptima Qual[a]	Aptima Quant[a]	cobas Qual[a]	Xpert VL-XC (POC)[b]	Xpert Qual-XC (POC)[b]
A2	0	NR	NR	NR	**R**	**114,719**	**R**	**50,500**	
	19	NR	NR	NR	**R**	**38,623**	**R**	**17,700**	
	29	NR	NR	NR	**R**	**1,606**	**R**	**865**	**Detected**
	40*	NR	NR	NR	**R**	**160**	**R**	**144**	
	**60**	NR	**R**	**R**	**R**	**1,528**	**R**	**958**	
A3	0	NR	NR	NR	**R**	**1,675**	**R**	**1,140**	
	14	NR	NR	**R**	**R**	**<30**	**R**	**<40**	
	28	NR	NR	NR	NR	ND	NR	ND	ND
	35*	NR	NR	NR	NR	ND	**R**	ND	
	42	NR	NR	NR	**R**	ND	NR	ND	
	65*	NR	NR	NR	NR	ND	NR	ND	ND
	**72**	NR	**R**	NR	NR	ND	NR	ND	
A4	0	NR	NR	**R**	**R**	**149,971**	**R**	**100,000**	
	17	NR	NR	NR	**R**	**<30**	**R**	**<40**	
	30	NR	NR	NR	NR	ND	NR	ND	ND
	38*	NR	NR	NR	NR	ND	NR	**<40**	
	45	NR	NR	NR	**R**	**<30**	QNS	**<40**	
	**63***	NR	**R**	NR	NR	**<30**	NR	**<40**	ND
C1	0	NR	NR	NR	**R**	**192**	**R**	**229**	ND
	9*	NR	NR	NR	**R**	**105**	**R**	**160**	
	17	NR	NR	NR	**R**	**39**	QNS	**123**	
	37*	NR	NR	NR	**R**	**3,278**	**R**	**3,290**	**Detected**
	**47**	NR	**R**	**R**	**R**	**2,598**	**R**	**2,660**	
C3	0	NR	NR	NR	**R**	QNS	QNS	QNS	
	8	NR	NR	NR	**R**	**<30**	QNS	**<40**	ND
	15*	NR	NR	NR	NR	**<30**	NR	**<40**	
	21	NR	NR	NR	**R**	**82**	QNS	**46**	
	**35**	**R**	**R**	**R**	**R**	**170,667**	**R**	**137,000**	**Detected**

Table 2B

Case[a]	Days since 1st HIV Pos[b]	Site test results (real-time)		HPTN Laboratory Center test results (retrospective)					
		Rapid Ab	Ag/Ab test	Ag/Ab test	Aptima Qual[a]	Aptima Quant[a]	cobas Qual[a]	Xpert VL-XC (POC)[b]	Xpert Qual-XC (POC)[b]
D1	0*	NR	NR	NR	**R**	**80**	**R**	**53**	ND
	14	NR	NR	NR	**R**	**139**	**R**	**95**	
	56*	NR	NR	NR	**R**	**65**	**R**	**58**	**Detected**
	77	NR	NR	NR	**R**	**60**	**R**	**<40**	
	**112***	NR	**R**	**R**	**R**	QNS	QNS	QNS	ND
D2	0	NR	NR	NR	**R**	QNS	QNS	QNS	
	42*	NR	NR	NR	NR	ND	NR	ND	ND
	55	NR	NR	NR	**R**	QNS	QNS	QNS	
	**98***	NR	**R**	NR	NR	QNS	QNS	QNS	ND
D3	0*	NR	NR	NR	**R**	**377**	**R**	**364**	**Detected**
	49	NR	NR	NR	**R**	ND	QNS	**<40**	
	62*	NR	NR	NR	**R**	**<30**	**R**	**53**	ND
	76	NR	NR	NR	**R**	**108**	**R**	**62**	
	**117**	**R**	**IND**	**R**	**R**	**8,049**	**R**	**4,260**	**Detected**
D4	0	NR	NR	NR	**R**	**<30**	QNS	Error	
	**45***	NR	**R**	**R**	**R**	**930**	QNS	**731**	**Detected**
D5	0	NR	NR	NR	**R**	**225**	QNS	Error	
	**42**	**R**	**R**	**R**	**R**	**23,283**	**R**	**21,900**	
D6	0*	NR	NR	NR	**R**	**2,282**	**R**	**2,240**	
	**59**	**R**	**R**	**R**	**R**	**13,521**	QNS	**9,580**	
BR1	0	NR	NR	NR	**R**	**468**	**R**	**379**	
	3*	NR	NR	NR	**R**	**99919**	**R**	**82,900**	
	31*	NR	NR	NR	**R**	**1670**	**R**	**2,710**	
	**81**	**R**	**R**	**R**	**R**	**222243**	**R**	**202,000**	

Footnotes for Table 2A and B.

[a] Reference test for this study.
[b] Assay under evaluation.

References

1. Marzinke MA, Grinsztejn B, Fogel JM, et al. Characterization of human immunodeficiency virus (HIV) infection in cisgender men and transgender women who have sex with men receiving injectable cabotegravir for HIV prevention: HPTN 083. J Infect Dis. 2021;224(9):1581-92.

2. Marzinke MA, Fogel JM, Wang Z, et al. Extended analysis of HIV infection in cisgender men and transgender women who have sex with men receiving injectable cabotegravir for HIV prevention: HPTN 083. Antimicrob Agents Chemother. 2023;67(4):e0005323.
